# Phenotypic characterization of an Arabidopsis T-DNA insertion line SALK_063500

**DOI:** 10.1016/j.dib.2018.03.107

**Published:** 2018-03-26

**Authors:** Natasha J. Sng, Anna-Lisa Paul, Robert J. Ferl

**Affiliations:** a Plant Molecular and Cellular Biology, University of Florida, Fifield Hall, 2550 Hull Road, Gainesville, FL 32611, USA; b Horticultural Science Department, University of Florida, Fifield Hall, 2550 Hull Road, Gainesville, FL 32611, USA; c Interdisciplinary Center for Biotechnology Research (ICBR), University of Florida, 2033 Mowry Road, Gainesville, FL 32601, USA

**Keywords:** Arabidopsis, T-DNA, SALK_063500, Silique, Pollen, Phenotype, AT1G05290

## Abstract

In this article we report the identification of a homozygous lethal T-DNA (transfer DNA) line within the coding region of the At1G05290 gene in the genome *of Arabidopsis thaliana* (Arabidopsis) line, SALK_063500. The T-DNA insertion is found within exon one of the AT1G05290 gene, however a homozygous T-DNA allele is unattainable. In the heterozygous T-DNA allele the expression levels of AT1G05290 were compared to wild type Arabidopsis (Col-0, Columbia). Further analyses revealed an aberrant silique phenotype found in the heterozygous SALK_063500 plants that is attributed to the reduced rate of pollen tube germination. These data are original and have not been published elsewhere.

**Specifications Table**

**Table:** 

Subject area	*Biology*
More specific subject area	*Plant biology*
Type of data	*Tables, Graphs, Figures*
How data was acquired	*DNA-PCR, Quantitative Realtime PCR (qPCR), Plant phenotypes, Pollen tube germination assay, Image J analyses*
Data format	*Raw, Analyzed*
Experimental factors	*Col-0 (Columbia) and SALK_063500 Arabidopsis plants*
Experimental features	*DNA-PCR was employed to identify the T-DNA insertion in SALK_063500. AT1G05290 expression levels were examined with qPCR. Both silique and pollen tube germination phenotypes were recorded.*
Data source location	*Gainesville, Florida, USA*
Data accessibility	*Data is within this article.*

**Value of the data**

• T-DNA insertion lines provide an important resource for genetic analyses in plant research, and SALK lines are the most commonly used T-DNA insertion lines. Therefore assessments of phenotypes observed in SALK lines are valuable assets for advancing our understanding of basic plant biology.
• Documentation of the phenotype of the SALK_063500 line will make the plant community aware of the role AT1G05290 plays in pollen development, thereby furthering research in this field.
• The data presented could provide insights into understanding the molecular mechanisms of male sterility in plants.

## 1 Data

**Fig. 1:**
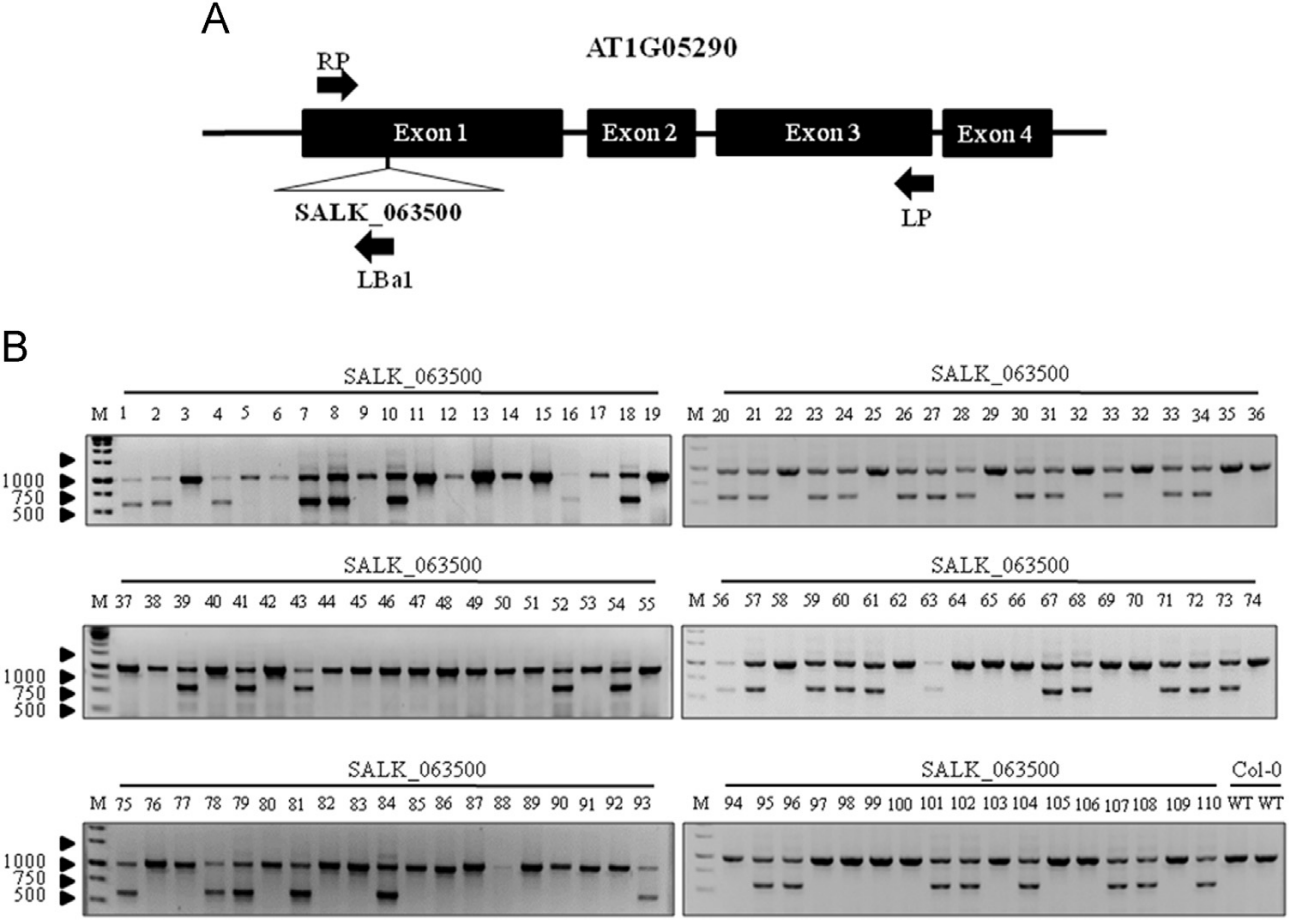
T-DNA insertion in the exon1 of AT1G05290 in SALK_063500. (A) Gene structure of AT1G05290. The T-DNA position is +127 bp after the transcription start site. Primers used to screen the SALK_063500 line, indicated by black arrows RP, LBa1, and LP, and were derived from the SALK T_DNA primer design web tool (http://signal.salk.edu/tdnaprimers.2.html). (B) PCR amplification of wild type allele band using forward (RP), reverse (LP) and T-DNA band (LBa1). 110 randomly selected SALK_063500 seedlings and two Col-0 wild type (WT) seedlings were used in search for a homozygous T-DNA insertion line, none were identified. Homozygous wild type allele are seen as single bands at 991 bp whereas heterozygous individuals have double bands, with a single wild type allele band at 991 bp and a T-DNA allele band at around 440–740 bp. All primer sequences are listed in Table 1.

**Fig. 2:**
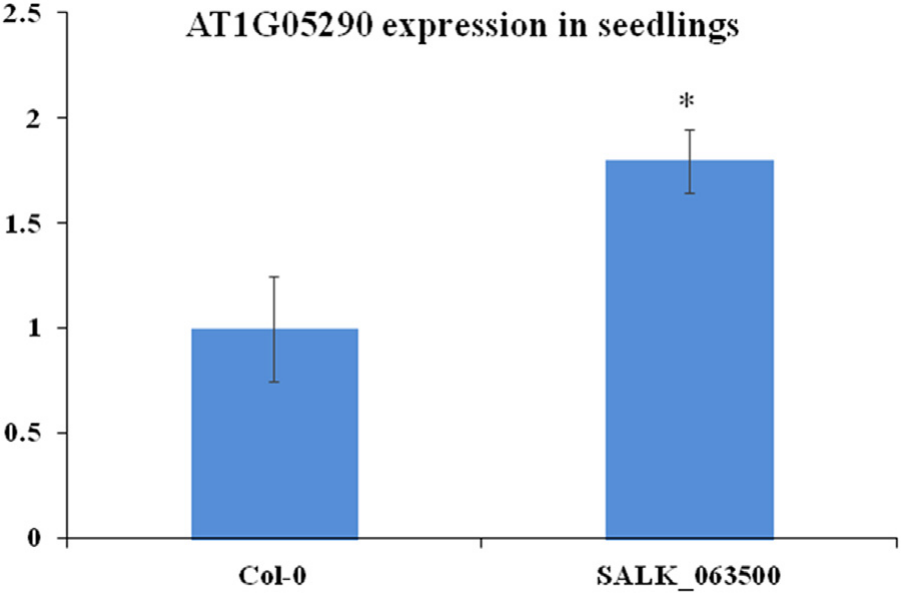
Relative expression levels of AT1G05290. The expression level of AT1G05290 in seedlings of Col-0 was set at “1” so that the relative expression level of SALK_063500 can be compared to Col-0. UBQ11 (AT4G05050) used as an internal control. The Ct (cycle threshold) values are shown in Supplementary table 1. Data represent means of standard error (SE) (*n* = 6). The student's *t*-test was performed to show the significant difference of gene expression between Col-0 and SALK_063500 (*p< 0.05).

**Fig. 3:**
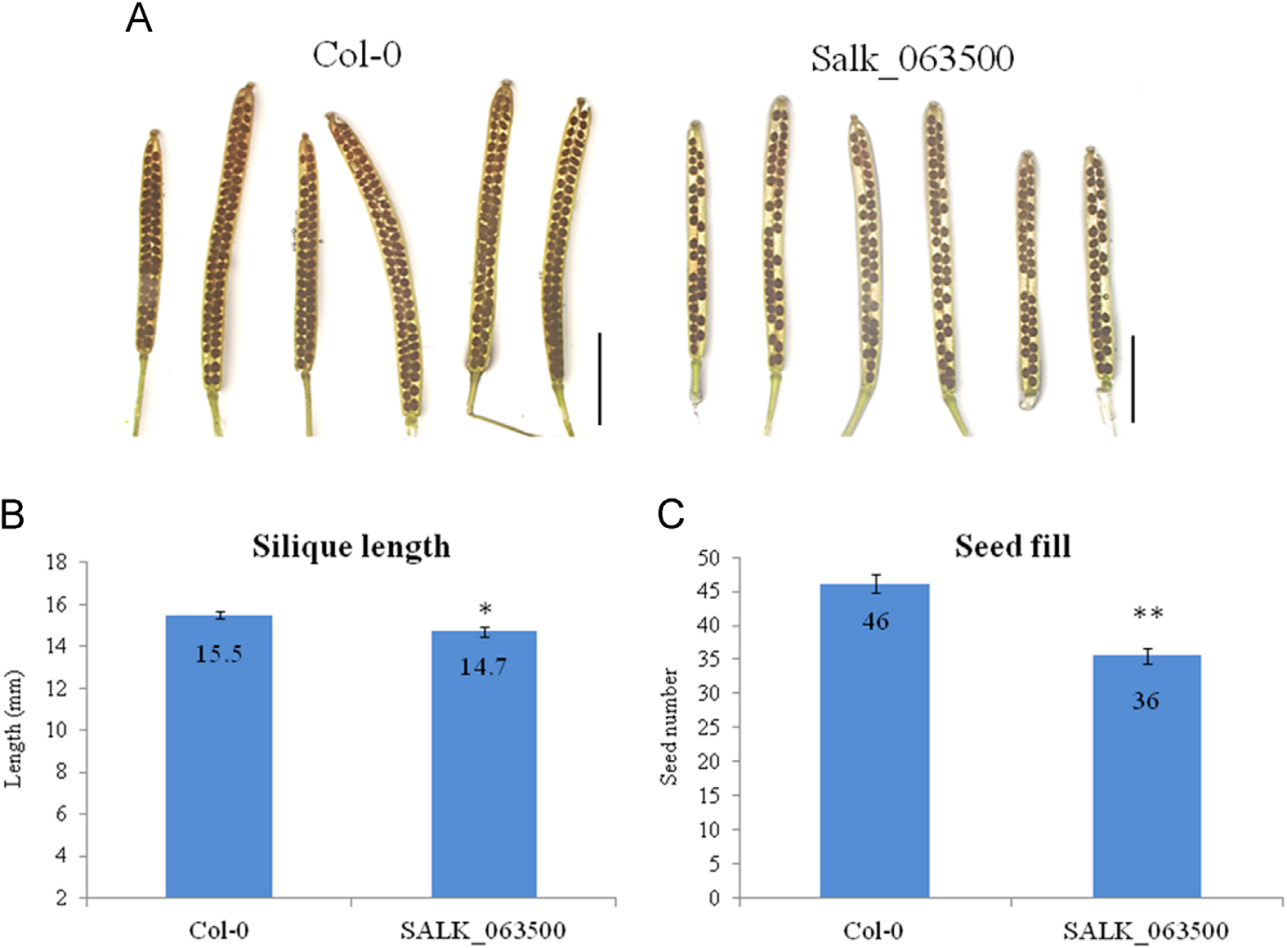
Phenotype of heterozygous SALK_063500 siliques. (A) Phenotype of wild type Col-0 and heterozygous SALK_063500 silique development. Scale bar = 5 mm. (B) Quantification of silique length and seed fill in Col-0 and heterozygous SALK_063500. The numbers of siliques used are shown in Supplementary table 2. Data presented are means±standard deviation (SD) (*n* = 30). The student's *t*-test was performed to show the significant difference of silique length and seed fill between Col-0 and heterozygous SALK_063500 (*p< 0.05, **p< 0.01).

**Fig. 4:**
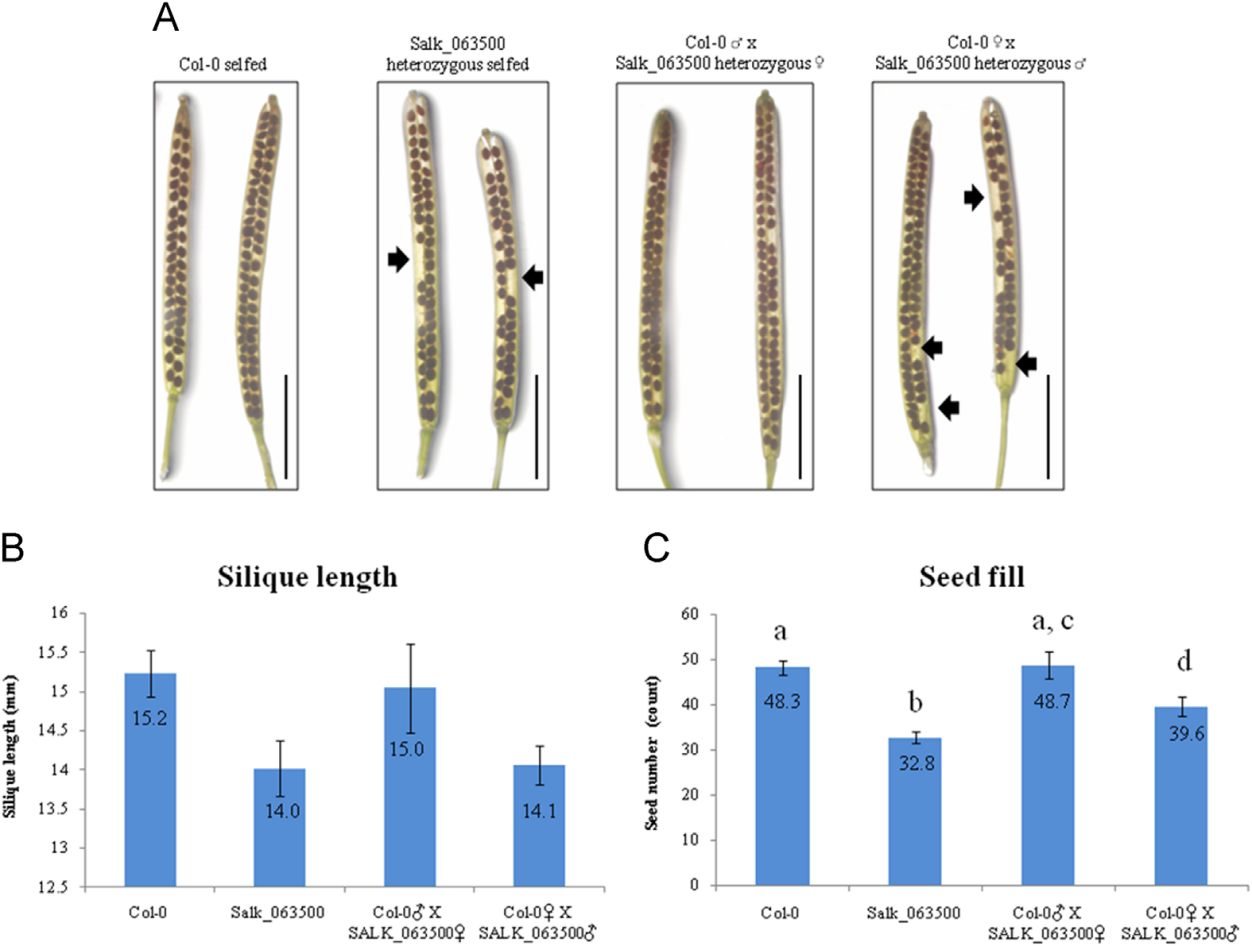
Phenotype of siliques from reciprocal cross between Col-0 and heterozygous SALK_063500 lines. (A) Phenotype of silique development in wild type Col-0, heterozygous SALK_063500, and their respective reciprocal crosses. Scale bar=5 mm. (B) The number of siliques used are shown in Supplementary table 3. Data presented are means±SD (*n* = 10). Both an ANOVA and the student's *t*-test was performed to show the significant difference of silique length and seed fill between all four lines (Supplementary table 3). Bar graphs with different letters show significant difference (*p* < 0.05).

**Fig. 5:**
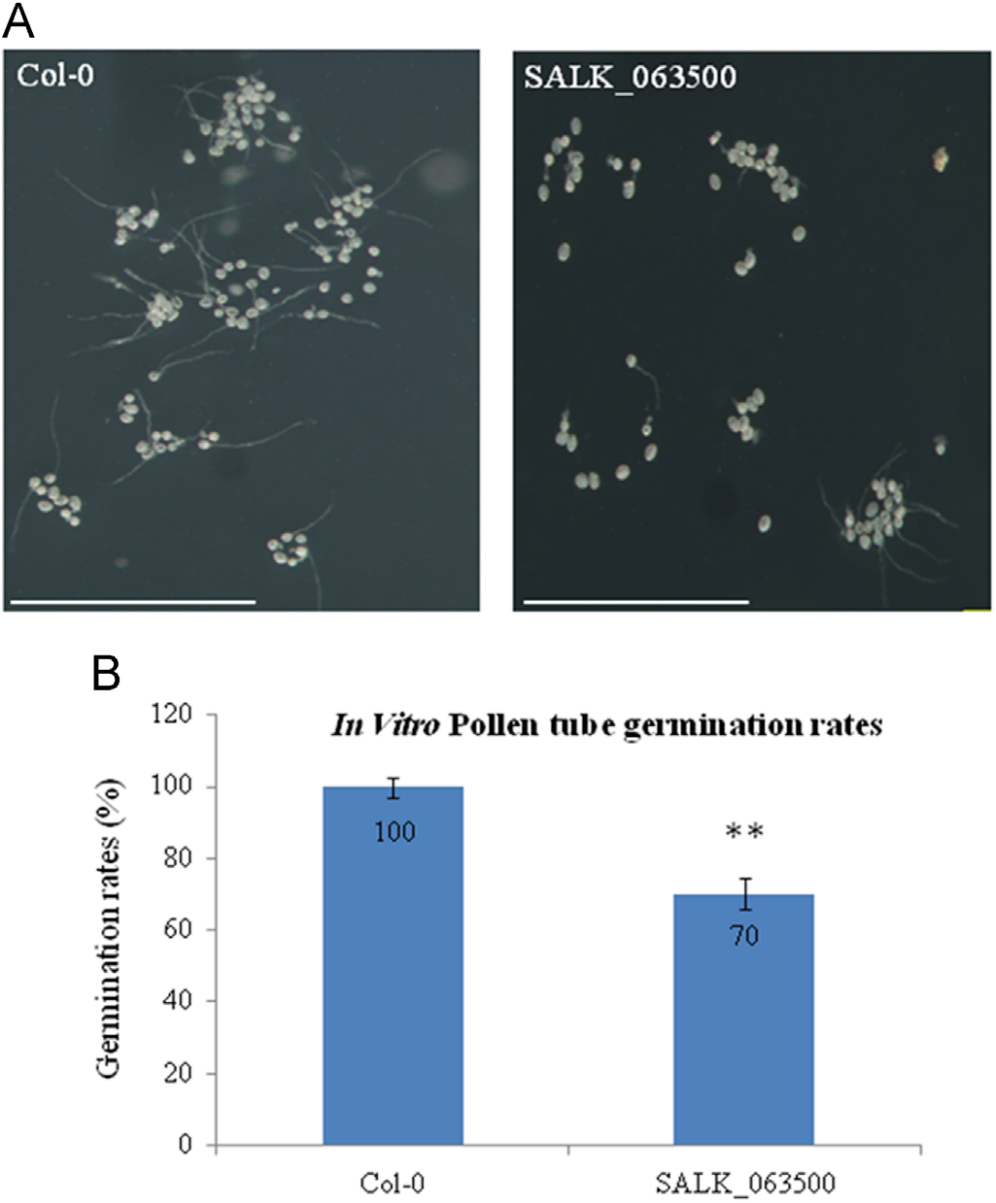
Pollen tube germination analysis *in vitro*. (A) *in vitro* pollen tube germination analysis of Col-0 and heterozygous SALK_063500 pollen grown overnight at room temperature on pollen germinating media. (B) Quantification of *in vitro* pollen tube germination of Col-0 and heterozygous SALK_063500 pollen. The numbers of pollen counted are shown in Supplementary table 4. Data presented are means±SD (*n* = 300). The student's *t*-test was performed to show the significant difference of *in vitro* pollen germination rates between Col-0 and heterozygous SALK_063500 (**p < 0.01).

**Fig. 6:**
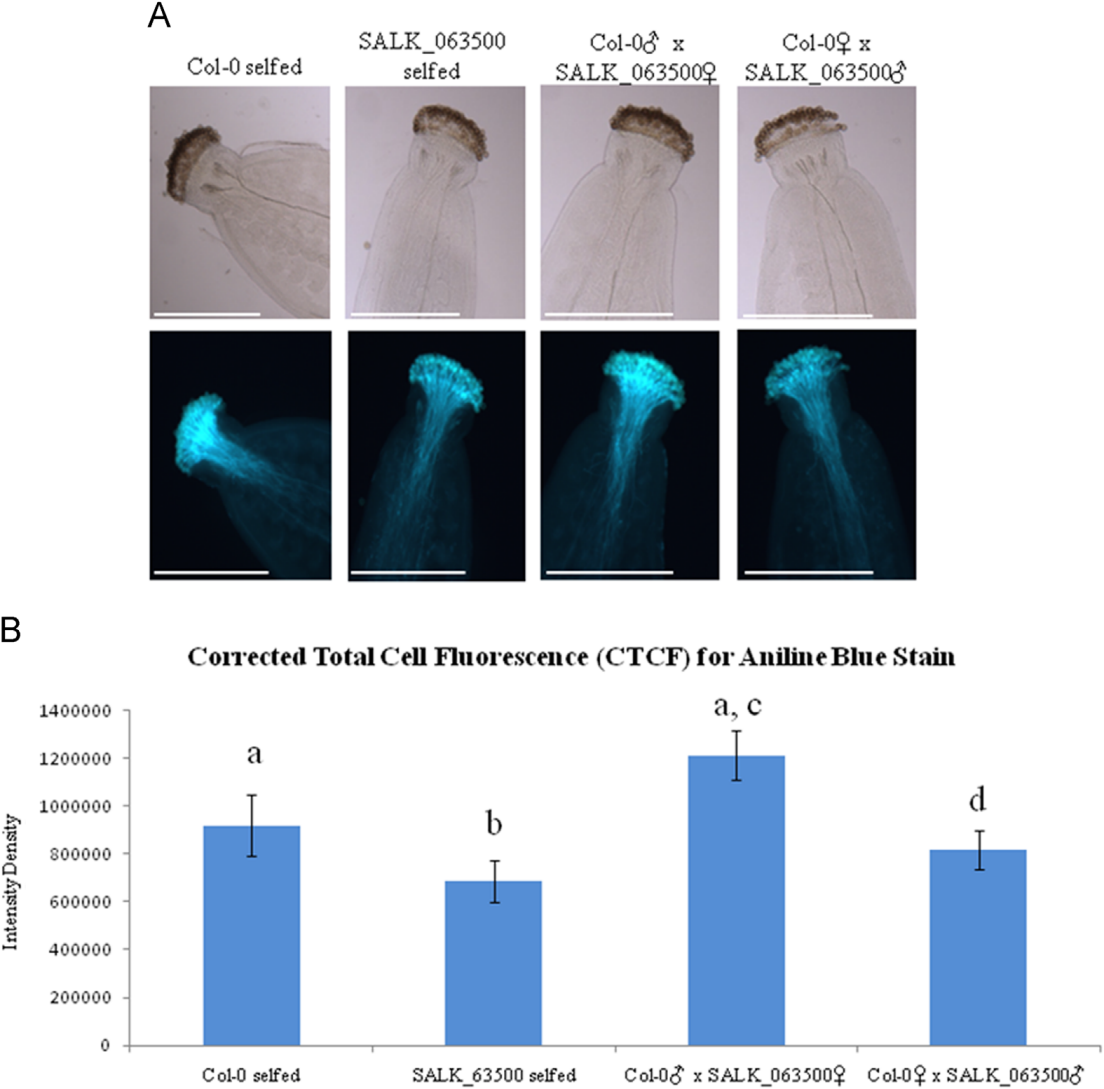
Pollen tube germination analysis *in vivo.* (A) Aniline Blue stain of Col-0, heterozygous SALK_063500 and, reciprocally crossed pollen tubes 6 h after pollination. Brightfield exposure was set at 3.59 ms and GFPA at 624 ms exposure. Scale bar = 500 um. (B) Quantification of the Corrected Total Cell Fluorescence (CTCF). Calculations for CTCF are shown in Supplementary table 5. Data presented are means±SD (*n* = 4). Both an ANOVA and the student's *t*-test was performed to show the significant difference of CTCF between all four lines. Bar graphs with different letters show significant difference (*p*< 0.05).

The data presented here provide information on the phenotype of a T-DNA insertion line, SALK_063500. Fig. 1A-B shows the T-DNA insertion within AT1G05290 and the wild type and heterozygous T-DNA alleles amplified. No homozygous T-DNA alleles were found after screening over a thousand SALK_063500 progeny. Fig. 2 shows the level of AT1G05290 expression in seedlings under normal growth conditions. Fig. 3, Fig. 4 show the silique phenotype observed in both Col-0 and heterozygous SALK_063500 plants. Fig. 5, Fig. 6 show the pollen tube germination rates *in vitro* and *in vivo* of Col-0 and heterozygous SALK_063500 plants. Table 1 provides the sequences of the primers used in Fig. 1, Fig. 2.

**Table 1:** Primers used in the data presented.

*Gene*	*Name*	*Sequence (5’-3’)*	*Usage*
AT1G05290	AT1G05290-LP	CGATCTTTCGGACTAAATCCC	T-DNA PCR
	AT1G05290-RP	AGACCGCTCTTGTAGAATCCC	
	AT1G05290-F	CTCACGGAGCTAATCGATGAATT	qPCR
	AT1G05290-R	CGATGACATACCAGATGCTGAAG	
N/A	LBa1	TGGTTCACGTAGTGGGCCATCG	T-DNA PCR
UBQ11 (AT4G05050)	UBQ11-F	AGCAACTTGAGGACGGCAGA	qPCR
	UBQ11-R	GTGATGGTCTTTCCGGTCAAA	

## 2 Experimental design, materials and methods

Arabidopsis seeds of wild type Columbia-0 (Col-0) and SALK_063500 were obtained from the Arabidopsis Biological Resource Center [1]. Plants were grown in the growth chambers on soil at 22 °C under constant light at approaximately 80 μmol m^−2^ s^−1^. In the search for a homozygous T-DNA insertion line from SALK_063500, over a thousand progeny of the heterozygous SALK_063500 line were screened and 110 are presented in Fig. 1B. AT1G05290-LP and AT1G05290-RP primers were used to amplify the wild type allele band (991 bp) while LBa1 and AT1G05290-RP primers were used to amplify the T-DNA band (440–740 bp). No homozygous T-DNA allele lines were found. For gene expression analysis, Col-0 and SALK_063500 seeds were grown on 0.5% Phytagel^TM^ plates (0.5× Murashige and Skoog (MS) salts [Invitrogen] containing 0.5% (w/v) sucrose, was adjusted to pH 5.72 with 1N KOH and solidified with 0.5% (w/v) Phytagel^TM^ [Sigma]) at 22 °C under constant light condition [2]. The 12 day old plants were harvested from Phytagel plates and directly preserved in RNALater [Ambion] until ready for RNA extraction. Total RNA was extracted using the RNAeasy kits [Qiagen] according to the manufacturer's instructions. CDNA was transcribed from total RNA, using the High Capacity RNA to cDNA kit [Applied Biosystems] and RT-qPCR analysis was conducted with SYBR Green master mix [Applied Biosystems]. The 2^−ΔΔCt^ method was used to calculate the relative expression of At1g05290 using *UBQ11* (AT4G0505) as an internal control. A total of 6 replicates were used for each sample. For silique phenotypes, 30 siliques from three individuals representing the genotype were collected, imaged under the Olympus stereo dissecting microscope (SZH10) and measured using image J [3], [4]. For *in vitro* pollen germination studies, mature pollen was harvested from flowers left at room temperate for 2 h, and then grown overnight on slides coated with solid pollen germinating media (18% Sucrose, 0.01% Boric Acid, 1 mM CaCl2, 1 mM Ca(NO3)2, 1 mM MgSO4 and 0.5% Noble agar [Difco] was adjusted to pH 7.0 [5]. Pollen germination studies done *in vivo* were performed by collecting pollinated pistils 6 h after pollination and briefly fixed in ethanol:acetic acid (3:1) for 2 h at room temperature. Fixed pistils were then washed with distilled water three times and further treated in 8 M NaOH overnight. Pistil tissues were then washed in distilled water and stained with aniline blue solution (0.1% aniline blue in 0.1 M K_2_HPO_4_-KOH buffer, pH 11) for 5 h in the dark before being viewed under the Olympus BX51 compound light microscope [6].
