# Supplementary material for: Phenotypic characterization of an Arabidopsis T-DNA insertion line SALK_063500
Source: Data Brief. 2018 Mar 26;18:913–9. doi: 10.1016/j.dib.2018.03.107 (PMC5996828; doi:10.1016/j.dib.2018.03.107)
Supplement: Supplementary file 1 — Supplementary material [file mmc1.docx]

Conflict of Interest

All the author declares no Conflict of Interest.
